# Integration of proteomic and metabolomic analyses: New insights for mapping informal workers exposed to potentially toxic elements

**DOI:** 10.3389/fpubh.2022.899638

**Published:** 2023-01-25

**Authors:** Alda Neis Miranda Araujo, Isabelle Nogueira Leroux, Danielle Zildeana Sousa Furtado, Ana Paula Sacone da Silva Ferreira, Bruno Lemos Batista, Heron Dominguez Torres Silva, Evangelos Handakas, Nilson Antônio Assunção, Kelly Polido Kaneshiro Olympio

**Affiliations:** ^1^Graduate Program in Translational Medicine, Paulista School of Medicine, Department of Medicine, Federal University of São Paulo, São Paulo, Brazil; ^2^School of Public Health, Department of Environmental Health, University of São Paulo, São Paulo, Brazil; ^3^Department of Chemistry, Institute of Environmental, Chemical, and Pharmaceutical Sciences, Federal University of São Paulo, Diadema, São Paulo, Brazil; ^4^Technology School of Teresina, Teresina, Piauí, Brazil; ^5^Center for Natural and Human Sciences, Federal University of ABC, São Paulo, Brazil; ^6^Department of Medicine, Computation and Medicine, Imperial College London, London, United Kingdom

**Keywords:** proteomics, metabolomics, exposome, potentially toxic elements, informal work, occupational exposure, multi-omics

## Abstract

Occupational exposure to potentially toxic elements (PTEs) is a concerning reality of informal workers engaged in the jewelry production chain that can lead to adverse health effects. In this study, untargeted proteomic and metabolomic analyses were employed to assess the impact of these exposures on informal workers' exposome in Limeira city, São Paulo state, Brazil. PTE levels (Cr, Mn, Ni, Cu, Zn, As, Cd, Sn, Sb, Hg, and Pb) were determined in blood, proteomic analyses were performed for saliva samples (*n* = 26), and metabolomic analyses in plasma (*n* = 145) using ultra-high performance liquid chromatography (UHPLC) coupled with quadrupole-time-of-flight (Q-TOF) mass spectrometry. Blood PTE levels of workers, controls, and their family members were determined by inductively coupled plasma-mass spectrometry (ICP-MS). High concentration levels of Sn and Cu were detected in welders' blood (*p* < 0.001). Statistical analyses were performed using MetaboAnalyst 4.0. The results showed that 26 proteins were upregulated, and 14 proteins downregulated on the welder group, and thirty of these proteins were also correlated with blood Pb, Cu, Sb, and Sn blood levels in the welder group (*p* < 0.05). Using gene ontology analysis of these 40 proteins revealed the biological processes related to the upregulated proteins were translational initiation, SRP-dependent co-translational protein targeting to membrane, and viral transcription. A Metabolome-Wide Association Study (MWAS) was performed to search for associations between blood metabolites and exposure groups. A pathway enrichment analysis of significant features from the MWAS was then conducted with Mummichog. A total of 73 metabolomic compounds and 40 proteins up or down-regulated in welders were used to perform a multi-omics analysis, disclosing seven metabolic pathways potentially disturbed by the informal work: valine leucine and isoleucine biosynthesis, valine leucine and isoleucine degradation, arginine and proline metabolism, ABC transporters, central carbon metabolism in cancer, arachidonic acid metabolism and cysteine and methionine metabolism. The majority of the proteins found to be statistically up or downregulated in welders also correlated with at least one blood PTE level, providing insights into the biological responses to PTE exposures in the informal work exposure scenario. These findings shed new light on the effects of occupational activity on workers' exposome, underscoring the harmful effects of PTE.

## 1. Introduction

Documented health effects of potentially toxic elements (PTEs) exposure include neurotoxicity, genotoxicity, carcinogenicity (1, 2), as well as enzyme inhibition (3, 4), harming of the central nervous system (CNS), energy metabolism, ion transporters, cardiovascular system, and mitochondrial dysfunction (5). Industrial activities, agrochemicals, and petroleum-derived products can all be sources of PTE exposure (6, 7). Some occupational exposure environments can be especially concerning, as they are found in a domiciliary setting and involve handling PTEs (8). Unfortunately, working under these conditions has been reported in many places around the world. Cottage industries in Africa are considered a public health problem, exposing entire families to high Pb levels (9, 10). Studies have reported concerning levels of Pb among jewelry workers in India and Pakistan (11–14), while in Brazil this scenario can be found in the Limeira municipality fashion jewelry production chain.

Welding and assembly are among the most frequently used processes in fashion jewelry production and represent major exposure sources of PTEs. Home-based and informal work in the fashion jewelry industry can lead to exposure environments that pose high risk to human health. During the assembly process, a fine-grained powder of material is released from the piece, and the welding process typically involves torches, cooking gas, welding powders (flux), wire and acid solutions (15, 16). The welding powder spreads throughout the household environment and the fumes produced can expose everyone in the vicinity, since personal protection equipment (PPE) is rarely used (16, 17). More sobering is the fact that this work activity sometimes takes place in the same room where food is prepared with children sharing the workspace (15, 16, 18, 19).

X-ray fluorescence analyses of the surface layer of the jewelry in these local production chains showed lead and cadmium concentrations that exceeded levels permitted by Brazilian legislation, besides high levels of other Potentially Toxic Elements (PTEs) unregulated by Brazilian law (nickel and tin). Cadmium, lead, and tin were also found in high concentrations in the welding powder used (15, 20). Values were also found to be above the Minimal Risk Level for chronic inhalation (MRL) for the elements Mn, Ni and Cd and environmental limits for Mn, Ni, Zn, Cd and Pb for levels determined on the welders' breathing zone. Furthermore, the measured concentrations of Cu, Zn, Cd, and Pb exceeded even the occupational limits (16).

Environmental and occupational exposures play an important role in an individual's exposome, constituting an external specific factor (21, 22). According to the top-down approach, designed to enable exposome studies, the toxic effects of external exposures, regardless of their starting point, are mediated by chemical compounds that change molecules, cells, and physiologic processes in the body (23, 24). Therefore, using omics technologies such as proteomics and metabolomics, it is now possible to assess both environmental compounds, found in very low levels of plasma, and the potential metabolic effects that precede a health diagnosis (21, 25).

Previous studies have used proteomics (26–29) and metabolomics (30–35) to investigate metabolic changes related to occupational PTE exposure. The present study aimed to apply proteomic and metabolomic analysis to access the impact of PTEs exposure on informal workers exposome from the fashion jewelry production chain in the city of Limeira, Sao Paulo state, Brazil.

## 2. Materials and methods

### 2.1. Study population

The study population was difficult to access. We relied on the assistance of the Limeira's Municipal Department of Health and Family Health Care Centers of the Brazilian Public Health System. The first contact with the families was facilitated by the local community health worker, who provided access to the participants by the researchers. A relationship of trust was built with the informal workers. The workers were then invited to take part in the study and subjects who agreed to participate signed the informed written consent form. Participants included in the exposure group were the workers engaged in the fashion jewelry production chain, irrespective of the activity they performed (assembling or welding), together with their relatives. The workers' relatives were also included in the wider study population, since they lived in the same environment where the work with jewelry was performed. Forty-six exposed families were invited, and 38 agreed to participate in the study. The control families invited lived near the exposed participants, but at least four houses away on the same street. For inclusion in the control group, no members of the family worked with jewelry or had known contact with chemical substances at work. Thirty-three control families were invited to take part in the study and 30 agreed to participate. However, between the time of scheduling and sample collection, 14 families dropped out. The final sample comprised 29 exposed families (total 112 participants including informal workers and relatives) and 23 control families including 53 participants with no exposure to chemical substances (16, 36). The biological samples (blood and saliva) were collected between July and August 2017. The study was approved by the institutional Ethics Committees of the School of Public Health of the University of Sao Paulo (Protocol Number 41965115.0.0000.5421) and the Federal University of São Paulo (Permit number 1459/2018), and all participants signed an informed consent term agreeing to take part in the study.

### 2.2. Sample collection and preparation

All biological samples were collected on the same day and after 4-hours fasting. The blood collection was performed by a trained nurse after the workers shifts for the exposed group and during the day for the control group. Blood samples were collected in heparinized tubes free of trace elements for PTE determination and in lithium heparin and separating gel for metabolomic tests (Vacutainer^®^). Saliva samples (2 mL) were collected using an adapted sputum method (37). Prior to collection, subjects lips were cleaned with gauze and water to avoid sample contamination.

After collection, saliva samples were homogenized in a universal collection tube (polypropylene) by adding 50 μL of formic acid (10 mmol L^−1^) and aliquoted (200 μL each aliquot) on the same day before storage at −80°C until processing and analysis. Metabolomic blood samples were also processed before storage. In this case, the tubes were centrifuged for 10 min at 4°C 2000 × g and the plasma aliquoted.

Blood samples for PTE determination were stored at −80°C until the chemical analysis. Before analysis, samples were diluted 1:50 in a 15 mL polypropylene Falcon^®^ tube (Becton Dickinson) with a solution containing 0.01% (v/v) Triton^®^ X-100, 0.5% (v/v) nitric acid and 10 μgL^−1^ of each respective internal standard (Yttrium). High purity de-ionized water (resistivity 18.2 MΩ cm^−1^) was used for preparing the samples and solutions (38).

### 2.3. Environmental exposure biomarkers

A total of 6 mL of whole blood were collected in heparinized tubes free of trace elements (Vacutainer^®^) to determine PTEs (Cr, Mn, Ni, Cu, Zn, As, Cd, Sn, Sb, Hg, and Pb). Trace elements were determined by an inductively coupled plasma-mass spectrometer (ICP-MS) at the Federal University of ABC (16). The ICP-MS is equipped with a reaction cell (ICP-MS 7900, Hachioji, Japan) and operated with high-purity argon (99.999%, White, Brazil) and helium (99.999%, White, Brazil) as the reaction gas. For quality control, the method was validated by analyzing certified reference material (CRM) for each batch of samples (Seronorm^®^ TE Whole Blood Level II – Stasjonsveien). The limits of detection (LOD) were 0.1597, 0.036, 0.164, 0.534, 1.060, 0.096, 0.001, 0.017, 0.005, 0.1507, and 0.002 μg L^−1^ for Cr, Mn, Ni, Cu, Zn, As, Cd, Sn, Sb, Hg, and Pb, respectively. The CRM recovery for all elements was 80–120%.

### 2.4. Saliva digestion- Proteomic measurements

Proteins were extracted based on the extraction protocol proposed by Friedman (39). First, 900 μL of chloroform 450 μL of methanol and 375 μL of water were added to saliva aliquot of 60 uL to precipitate the proteins, and their concentration was then determined using a BCA test kit (BioRad, USA) (40). This process was repeated until to achieve 100 μg of extracted proteins, which then were enzymatically digested (as described in Supplementary material) and analyzed with a nanoACQUITY UPLC^®^ System coupled with a mass spectrometry system (maXis™ 3G Q-TOF), in single shotgun runs. MS/MS spectra were analyzed using the software PEAKS studio 8.5 (Bioinformatics Solutions Inc., Canada) and the Uniprot (February 2019) database was used for Homo sapiens taxonomy (20,415 entries). Saliva samples were processed by a peak list (MGF format) and analyzed together for protein identification with a False Discovery Rate (FDR) cut-off of 1%, protein score identification (−10l gP) of ≥20, and single peptide > 15 (41). The other parameters are specified in the Supplementary material.

### 2.5. Untargeted metabolomic measurements

Blood plasma samples were collected and analyzed using a UHPLC Agilent 1290 Infinity II (Agilent Technologies) device coupled with a high-resolution QqTOF mass spectrometer (Bruker Daltonics - Impact, Rheinstetten, Germany). Details are provided in the Supplementary material.

### 2.6. Statistical analysis, bioinformatics, and gene ontology

The statistical analysis of the PTE levels was performed using Statistica 12.5. A non-paired two-tailed Student's *t*-test was performed to identify the metals whose concentrations differed between the Welder and Control groups.

**Figure 1:**
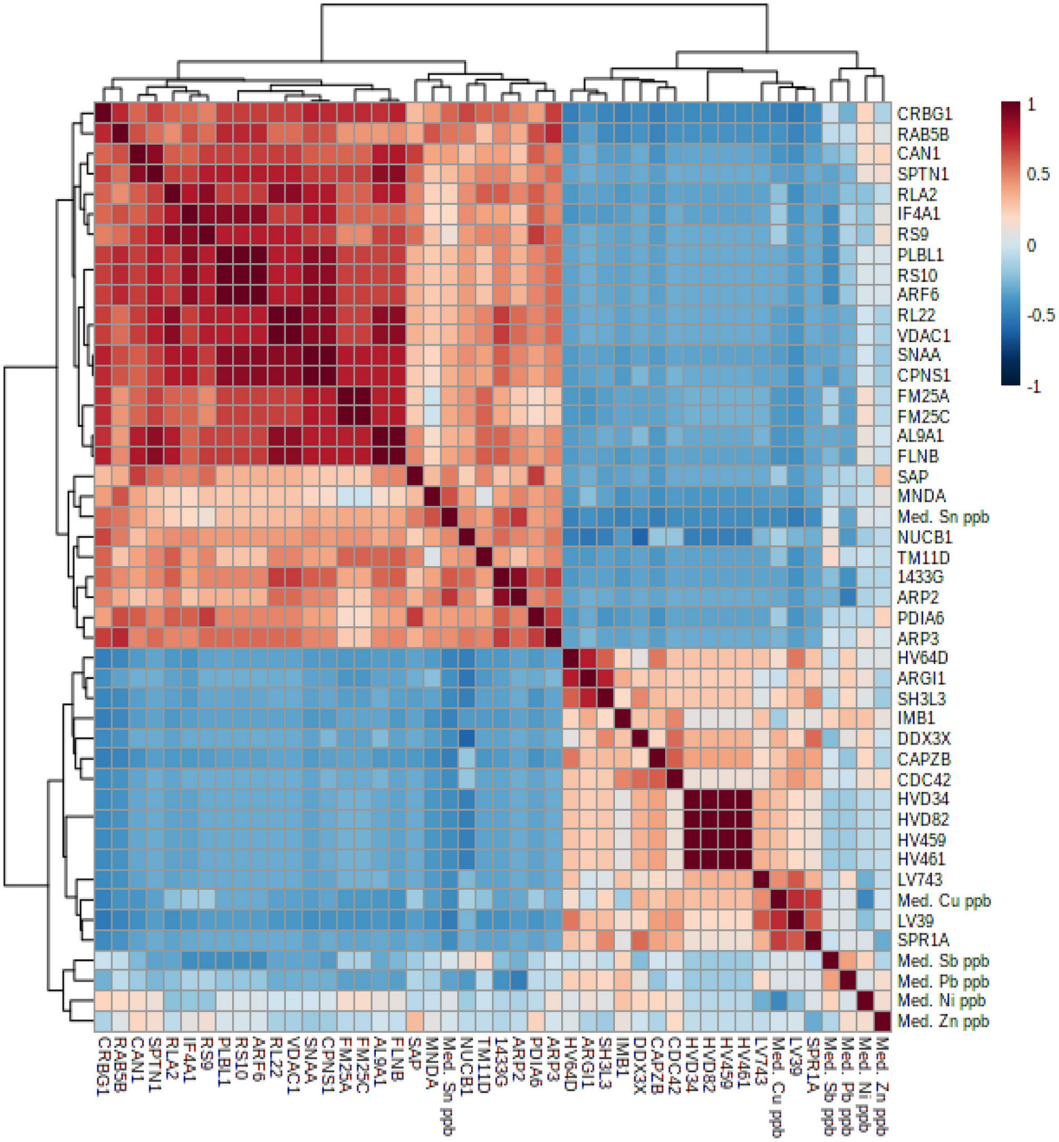
Correlation heatmap with hierarchical cluster analysis based on Pearson correlation coefficient. Distance is scaled and values range from 1 (red color) to −1 (dark blue color). Correlation of gene saliva expression associated with blood Ni, Cu, Zn, Sn, Sb, and Pb metal concentrations.

Statistical analysis of the proteins was conducted on MetaboAnalyst 4.0 (42) using the 979 proteins identified in both study groups. Of the total 979 proteins, those with missing values in 50% or more samples were excluded, giving 411 proteins. The missing values were due to the equipment resolution. The missing values for the 411 remaining variables were estimated by the k-nearest neighbors' algorithm (k-NN). The data were subsequently normalized by automatic scaling. Principal components analysis (PCA), Partial Least-Squares Discriminant Analysis (PLS-DA), and Student's *t*-test were then performed. The PCA unsupervised test was run in order to observe the tendency of the group separation. PLS-DA was then performed in order to check whether the proteins profile was able to distinguish between exposure and control groups (Supplementary Figure 2). A heatmap was constructed with the significant variables obtained from Student's *t*-test. Pearson's correlation between PTE levels and protein was determined and clusters identified among genes, saliva expression and blood Ni, Cu, Zn, Sn, Sb, and Pb levels based on Euclidean distance using MetaboAnalyst 4.0 for both analyses (42) (Figure 1).

Gene ontology consists of an instrument for biological study of genes (43). The bioinformatic tool DAVID (https://david.ncifcrf.gov/tools.jsp) (2021 update) was used for this analysis in order to determine the biological processes, molecular functions and cellular components encompassed by the proteins identified on Uniprot (44, 45).

A Metabolome-Wide Association Study (MWAS) was conducted to investigate the association between blood metabolomics and exposure groups using multiple linear regression models using R4.0.3. The dependent variable in the model was metabolite, and the exposure variable was exposure status (exposure and control groups). The model was adjusted for sex and age (46).

Then using the results of the MWAS analysis, a pathway enrichment analysis was conducted using the Mummichog program (47), supplemented with manual curation of the metabolite identities assigned by Mummichog, using a similar procedure to that adopted by Handakas (Supplementary material) (48). Detailed, the results of the MWAS were used as an input data from the mummichog. Mummichog requires two lists of m/z features, the significant list, and the reference list. Then, mummichog computes all possibly matched metabolites, and searches the reference metabolic network for all the modules that can be formed by these tentative metabolites.

Finally, a multi-omics (proteomics and metabolomics) analysis was performed to assess the metabolic pathways potentially disturbed by home-based occupational exposure to PTEs using MetaboAnalyst 4.0 (42).

## 3. Results

### 3.1. Study design

Supplementary Table 3 shows the characteristics of the population included in the present analysis. The subsample of participants for the proteomics study was selected based on the results from our previous study (16) when we evaluated the blood PTEs levels. In this study, we did not find a significant difference in PTEs levels between the groups when the wider population was included. But, the measured concentrations of Cu, Zn, Cd, and Pb exceeded the occupational limits. Values were also found be above the Minimal Risk Level for chronic inhalation (MRL) for the elements Mn, Ni and Cd and environmental limits for Mn, Ni, Zn, Cd and Pb for levels determined on the welders' breathing zone. As can be verified in the Supplementary Figure 1, the sample power analysis determines the minimum sample size of 27 participants needed for a statistical power of 0.8 and significance level of 0.05. Therefore, the sub-sample was selected on the basis of the results of our previous study, the high cost of performing the proteomics analysis, and the power analysis described above. A total of 26 participants were selected, comprising 13 welders and 13 controls, all women and within a similar age range. The metabolomics sample is larger and more diverse, as in other studies published by our research group (16, 36). The exposure group consists of jewelry workers and their relatives.

### 3.2. Proteomic results

Student's *t-*test and PLS-DA analysis were performed to search for proteins with levels that differed statistically between welders and controls. The target proteins found to be significant by Student's *t*-test (*p* < 0.05), corrected with the FDR (FDR <0.05), and that also had a Variable Importance in the Projection (VIP) score > 1.0, were considered significant proteins whose saliva levels were able to distinguish between the welders and control subjects. The predictive ability of this model (*Q*^2^) was 0.73, and the goodness of fit (*R*^2^) was 0.93. Protein regulation was defined by Student's *t-*test, in which significant concentrations were displayed on boxplots with their interpretation (whether up or downregulated in welders group) described in Table 1. Of the 40 proteins identified, 26 were upregulated and 14 downregulated in the welder group relative to the control group (Table 1).

**Table 1:** Proteins up or down-regulated in welders.

**Protein IDs**	**Gene names**	**Up/Down**
1433G	Tyrosine 3-monooxygenase/tryptophan 5-monooxygenase activation protein gamma (YWHAG)	Up
1433G	Tyrosine 3-monooxygenase/tryptophan 5-monooxygenase activation protein Gamma (YWHAG)	Up
AL9A1	Aldehyde dehydrogenase 9 family member A1 (ALDH9A1)	Up
ARF6	ADP ribosylation factor 6 (ARF6)	Up
ARGI1	Arginase 1 (ARG1)	Down
ARP2	ARP2 actin related protein 2 homolog (ACTR2)	Up
ARP2	ARP2 actin related protein 2 homolog (ACTR2)	Up
ARP3	ARP3 actin related protein 3 homolog (ACTR3)	Up
CAN1	Calpain 1 (CAPN1)	Up
CAN1	Calpain 1 (CAPN1)	Up
CAPZB	Capping actin protein of muscle Z-line beta subunit (CAPZB)	Down
CDC42	Cell division cycle 42 (CDC42)	Down
CPNS1	Calpain small subunit 1 (CAPNS1)	Up
CRBG1	Absent in melanoma 1 (AIM1)	Up
CRBG1	Absent in melanoma 1 (AIM1)	Up
DDX3X	DEAD-box helicase 3, X-linked (DDX3X)	Down
FLNB	Filamin B (FLNB)	Up
FLNB	Filamin B (FLNB)	Up
FM25A	Family with sequence similarity 25 member A (FAM25A)	Up
FM25C	Family with sequence similarity 25 member C (FAM25C)	Up
HV459	Immunoglobulin heavy variable 4-59 (IGHV4-59)	Down
HV461	Immunoglobulin heavy variable 4-61 (IGHV4-61)	Down
HV64D	Immunoglobulin heavy variable 3-64D	Down
HVD34	Immunoglobulin heavy variable 4-30-4 (IGHV4-30-4)	Down
HVD82	Immunoglobulin heavy variable 4-38-2 (IGHV4-38-2)	Down
IF4A1	Eukaryotic translation initiation factor 4A1 (EIF4A1)	Up
IMB1	Karyopherin subunit beta 1 (KPNB1)	Down
LV39	Immunoglobulin lambda variable 3-9 (gene/pseudogene) (IGLV3-9)	Down
LV39	Immunoglobulin lambda variable 3-9 (gene/pseudogene) (IGLV3-9)	Down
LV743	Immunoglobulin lambda variable 7-43 (IGLV7-43)	Down
LV743	Immunoglobulin lambda variable 7-43 (IGLV7-43)	Down
MNDA	Myeloid cell nuclear differentiation antigen (MNDA)	Up
NUCB1	Nucleobindin 1 (NUCB1)	Up
PDIA6	Protein disulfide isomerase family A member 6 (PDIA6)	Up
PLBL1	Phospholipase B domain containing 1 (PLBD1)	Up
RAB5B	RAB5B, member RAS oncogene family (RAB5B)	Up
RAB5B	RAB5B, member RAS oncogene family (RAB5B)	Up
RL22	Ribosomal protein L22 (RPL22)	Up
RLA2	Ribosomal protein lateral stalk subunit P2 (RPLP2)	Up
RS10	Ribosomal protein S10 (RPS10)	Up
RS9	Ribosomal protein S9 (RPS9)	Up
SAP	Prosaposin (PSAP)	Up
SH3L3	SH3 domain binding glutamate rich protein like 3 (SH3BGRL3)	Down
SNAA	NSF attachment protein alpha (NAPA)	Up
SPR1A	Small proline rich protein 1A (SPRR1A)	Down
SPR1A	Small proline rich protein 1A (SPRR1A)	Down
SPTN1	Spectrin alpha, non-erythrocytic 1 (SPTAN1)	Up
TM11D	Transmembrane protease, serine 11D (TMPRSS11D)	Up
VDAC1	Voltage dependent anion channel 1 (VDAC1)	Up

PCA analysis was carried out to observe the separation tendency between the exposure groups. In the present analysis, the first two principal components explained 26.4% of the protein level variance. The PCA score plots and PLS-DA showed a distinct separation between the two exposure groups (Supplementary Figure 2).

### 3.3. Gene ontology: Protein functional analysis

The gene ontology analysis of the 40 proteins found to exhibit a significant difference (*p*-value <0.05 and VIP score > 1.0) between exposed and control groups designates which biological processes, molecular functions or cellular components are regulated by these proteins.

The gene ontology results for the proteins upregulated in the welders are given in Supplementary Table 4. Some of the biological processes regulating these proteins include translation initiation, signal recognition particle (SRP)-dependent co-translational protein targeting to membrane, and viral transcription. Regarding the molecular functions of these proteins, the most significant terms were structural constituent of ribosome and protein binding. The main ontology terms of cellular components regulated by the upregulated proteins were extracellular exosome, focal adhesion and membrane.

The gene ontology results for the significant proteins found to be downregulated in older individuals are given in Supplementary Table 5. Some of the biological processes regulated by these proteins are the Fc-gamma receptor signaling pathway involved in phagocytosis and negative regulation of protein complex assembly. The only molecular function found to be significant for the downregulated proteins was antigen binding, and the cellular components were extracellular exosome and cytoplasm.

### 3.4. Blood PTE levels and correlated target proteins

The PTEs determined in blood samples are presented by exposure group in Supplementary Tables 6, 7. The welders exhibited higher blood Cu levels (*p* < 0.001). The mean blood Sn was not calculated for control subjects because most values found were below the Limit of Quantification, whereas the mean Sn value for exposed participants was 1.218 ppm. Therefore, it can be inferred that the welders may be exposed to this element throughout the working process.

**Figure 2:**
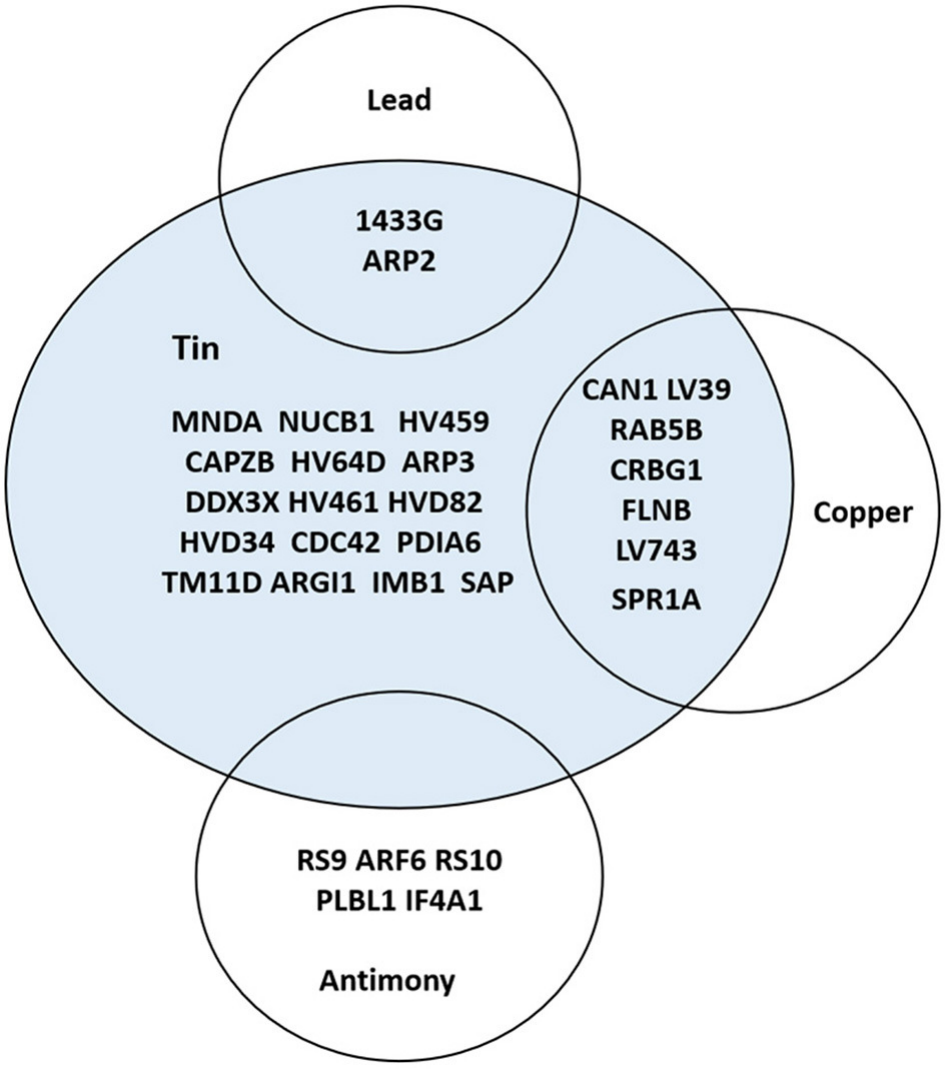
VENN diagram depicting saliva proteins correlated with Ni, Cu, Zn, Sn, Sb, and Pb levels (*p* < 0.05).

Hierarchical cluster analysis based on Pearson correlation coefficient was performed to correlate saliva gene expression with blood PTE levels. A total of 30 proteins were found to be significantly correlated (*p* < 0.05) with PTEs (Supplementary Table 8 and Figure 1), 14 of which were significantly correlated with two PTEs (Figure 2).

The analysis showed two clusters each with one subcluster. The first cluster consisted of 26 proteins upregulated in welders. The respective subclusters are formed by some proteins correlated with PTEs and others which are not. The second cluster consisted of 14 proteins downregulated in welders and the subcluster comprises proteins which are both downregulated and associated with PTE levels.

### 3.5. Blood PTE levels and correlated metabolites

The PTEs determined in blood samples are presented by exposure group in Supplementary Tables 6, 7. We evaluated the association between the blood biomarkers of exposure and metabolites with MWAS: 103 metabolic features were found significantly associated with the Cu blood levels and one feature with Zn levels. Then, those metabolites were used in the pathway enrichment analysis performed with the mummichog algorithm. That analysis found 118 metabolic pathways, 3 of them significant (*p* < 0.005), and with four or more of those metabolic compounds overlapped on the same pathway.

### 3.6. Metabolome-wide association and pathway enrichment analysis

Mummichog analysis indicated enrichment among exposure group and control group in the “Purine metabolism”, “Aspartate and asparagine metabolism”, and “Valine, leucine, and isoleucine degradation” pathways (Supplementary Table 1).

Additionally, for the subsample population of the 26 participants, Mummichog analysis indicated enrichment among exposure group and control group in the Arginine and Proline Metabolism, Carnitine shuttle, Purine metabolism, Urea cycle/amino group metabolism, Glycerophospholipid metabolism, Valine, leucine and isoleucine degradation, Aspartate and asparagine metabolism, Vitamin E metabolism, Prostaglandin formation from arachidonate, Pyrimidine metabolism, Glycine serine alanine and threonine metabolism information (Supplementary Table 2).

### 3.7. Joint multi-omics analysis of pathways

A joint analysis of pathways was performed of the 40 proteins up or downregulated in welders' saliva and the 73 significant metabolites associated with exposure status of the participants. The joint analysis of pathways revealed seven significant metabolic pathways (Supplementary Table 2).

## 4. Discussion

To the best of our knowledge, this is the first study investigating changes in metabolite and protein profiles of informal workers engaged in the jewelry production chain. As will be further detailed below, the proteins CRBG1, RAB5B, 1433G, MNDA, and RLA2 were found to be correlated with PTE levels in humans. These findings are novel and have not been described elsewhere. A total of 40 proteins were found to be differentially expressed in welders relative to controls: 26 upregulated and 14 downregulated in the workers (Table 1). Of the 26 upregulated proteins, 17 were correlated with at least one blood PTE level. Moreover, 14 proteins were found to be downregulated in the workers relative to controls, 13 of which were also correlated with blood PTE levels. Occupational exposure to harmful substances has been associated with a wide range of proteins involved in health outcomes (26, 27, 49). Each of the PTEs that the studied population may be exposed to has distinct toxicity mechanisms and can disturb gene expression of a protein in different cells or biological systems (50–56).

Blood sample analysis for proteomics is challenging. Blood encompasses the most complex human proteome (57), constituting 90% high-abundance proteins (such as albumin and IgG) (58). Given that the sample complexity results in loss of information on important proteins (59), we chose to investigate the saliva proteome, which is more homogeneous and requires fewer preparation steps, thereby minimizing identification and quantification errors (60–62).

Previous studies have used saliva as a biological matrix for biochemical assessment of human toxic exposure. Saliva is considered “the body mirror” as it has proteins of low abundance and great possibility for diagnosis (63–65), favoring the use of this matrix in exposome studies (62).

We found that the 1433G protein was upregulated in welders (Table 1) and correlated with blood Pb and Sn levels (Supplementary Table 8). The 1433G protein plays a role in signal transduction apoptosis and cell cycle regulation and is also used as a biomarker of neurological disorders, being involved in neural transmission regulatory processes and signal transduction (66–68). Xie et al. (69) reported that the 1433G protein was upregulated in a mushroom capable of accumulating Pb and Cd. In the present study, we failed to observe a significantly higher Pb concentration level in the exposure group compared to the control group (*p* = 0.053) (Supplementary Table 6). Nevertheless, in our recent study of the same Limeira population, we showed that blood Pb levels were determined in welders' breathing zone (16). Additionally, Salles et al. (70) found a higher Pb concentration in blood samples collected from a larger number of informal welders in the same municipality, in Brazil.

In the present study, higher blood Cu levels (*p* < 0.001) (Supplementary Table 6) were detected in welders compared to controls. In a previous study, we measured high levels of Cu in the welding supplies used by the same group of workers (36). At low levels, Cu is an essential compound for several enzymatic activities (71), but at high levels becomes toxic, accumulating in hepatocytes and inducing lipidic disturbance and chromosome 13 disorder (72, 73). Therefore, this element can be harmful to the kidneys and liver (74, 75) and is also associated with anemia, immunotoxicity and developmental impairment (76, 77). The present analysis revealed that seven proteins, including CRBG1, were correlated with blood Cu levels (Supplementary Table 8). The table (Supplementary Table 8) contains the interpretation of Pearson's correlation coefficient for each metal. Results show that CRBG1 and Sn are positively correlated while CRBG1 and Cu negatively correlated. However, CRBG1 was upregulated in the welders' group. Since CRBG1 was both positively correlated with Sn blood levels and upregulated, the higher the Sn level, the higher the CRBG1. Likewise, the negative correlation of CRBG1 with Cu indicates these variables are inversely correlated. Ray et al. (78) showed that CRBG1 was associated with melanoma and plays a suppressing role in malignant melanoma (78). RAB5B was another protein upregulated in the welders (Table 1) and correlated with blood Cu and Sn levels (Supplementary Table 8). The protein has previously been associated with sulfite oxidase deficiency and Parkinson disease and is a therapeutic target in Polycystic Ovary Syndrome (79).

The exposed group also had higher blood Sn levels, probably due to welding activity, given that most of the control participants had this trace element in amounts below the quantification limit. The PTE Sn had the highest number of proteins correlated with it, totaling 25 (Supplementary Table 8), comprising 12 upregulated and 13 downregulated in the welders (Table 1). MNDA and PDIA6 were 2 of the upregulated proteins (Table 1). The MNDA protein is a salivary biological marker for Sjogren Syndrome and for distinguishing between different kinds of lymphomas (80, 81). MNDA is also related to NF-kappa-B, apoptosis and autophagy signaling pathways. PDIA6 has an isomerase function (82) and its overexpression has been correlated with a poor cancer prognosis and serves as a biomarker for non-small cell lung cancer (NSCLC) (83). RLA2, another protein found to be upregulated in the welders (Table 1), has also been described as a marker of osseous metastasis and of other malignant tumors, such as gynecologic tumors and carcinoma of the pancreas, colon, and digestive system (84–86). RLA2 plays a role in protein synthesis lengthening, DNA repair, proliferation, apoptosis, and tumorigenesis. Pathways of this protein are related to viral mRNA translation and flu viral replication and transcription (86).

Monastero et al. (87) points out that the interpretation of changes in protein expression as a health effect of PTE exposure poses a challenge. The author also highlights that the study of the biological pathways is necessary in order to better understand which protein pathways are being up or downregulated. We performed a joint analysis of pathways for significant proteins and metabolites, identifying seven pathways that may be disturbed by PTE exposure. One of those pathways (arginine and proline metabolism; Table 2) have been reported to be impacted by Cd exposure through an analysis of the urinary metabolome of mice (88). Although the blood Cd levels detected in the present study were not significant, other studies conducted by our group in the same population have found high Cd levels in urine, as well as in welding powder (flux) and costume jewelry pieces (36).

**Table 2:** Statistically significant metabolic pathways from joint multi-omics analysis.

**Pathways**	**Raw *p*-value**	**FDR**
Valine, leucine, and isoleucine biosynthesis	1.01^−9^	3.33^−7^
Arginine and proline metabolism	4.57^−8^	7.57^−6^
ABC transporters	7.21^−7^	7.96^−5^
Arachidonic acid metabolism	1.42^−6^	1.14^−4^
Central carbon metabolism in cancer	4.53^−6^	3.00^−4^
Valine, leucine, and isoleucine degradation	3.31^−5^	1.82^−3^
Cysteine and methionine metabolism	9.44^−5^	4.46^−3^

The ABC transporters pathway has been previously implicated in heavy metal transportation, as well as metabolic diseases, cancer, and Alzheimer's disease (89). In this study, pathway activity was enriched in 3 and 11 pathways for the total population and the subsample, respectively. Although not based on fully validated annotation of the metabolome, Mummichog results express the likelihood of the involvement of specific metabolic pathways in the response to PTE exposure levels. Two out of the three pathways selected by Mummichog are related to amino acids for the total population. Additionally, the multi-omics pathway analysis in MetaboAnalyst showed that 3 out of the 11 metabolic pathways are related to amino acids (Supplementary Table 2). A metabolomic study of workers exposed to welding fumes also showed disturbance in amino acid pathways (leucine, isoleucine, and proline metabolism) (90). The nephrotoxicity of Cu nanoparticles in rats leads to disturbance in the expression of genes involved in valine leucine and isoleucine degradation (91).

Also, given the disrupted pathways are disturbed for both the blood metabolome and saliva proteome, use of both matrices increases the reliability of result, as both tissues validate the findings. The two analyses differ in terms of extraction methods, analysis equipment, and laboratory used. Therefore, final integration, i.e., analyzing each omics separately and combining their final predictions (92) may serve to validate the study findings regarding disturbed biological responses to occupational exposure. The large amount of information and limited success of traditional approaches justify the need for holistic approaches in an effort to obtain more consolidated results (93).

The present study has some limitations, such as the small number of participants for proteomic analysis, providing only preliminary results. Also, only workers who performed welding were included in the analysis, i.e., a specific sub-sample with higher chemical exposure in comparison with assembly workers. Furthermore, it is extremely hard to engage this population in research projects, as they have an informal occupation and are worried about losing their jobs. Another reason for the small number of participants included in the proteomic analyses is the difficulty for some participants to collect a sufficient amount of saliva. This situation arises mainly because of individual traits, but may also stem from the PTE exposure itself, whose presence has been reported to impair saliva secretion volume (44). Although potential biomarkers were found, the proteins were not confirmed by western blotting analysis in this phase of the study.

We have described metabolic and proteomic profiles associated with PTE exposure, highlighting the role of multiple metabolites, proteins, and genes in various pathways. Our findings shed new light on the effect of occupational activity on the workers' exposome, underscoring the harmful effects of PTE. These associations should be validated in future causal studies.

## Data availability statement

The data is available at https://zenodo.org/record/7510582#.Y7izcHbMJD8; doi: 10.5281/zenodo.7510582.

## Ethics statement

The studies involving human participants were reviewed and approved by the Ethics Committees of the School of Public Health of the University of São Paulo (Protocol Number 41965115.0.0000.5421) and the Federal University of São Paulo (Permit number 1459/2018). Written informed consent to participate in this study was provided by the participants' legal guardian/next of kin.

## Author contributions

AA and IL: methodology, investigation, formal analysis, and writing—original draft. DF, HS, EH, and AF: formal analysis and writing—review and editing. BB: formal analysis, resources, and writing—review and editing. NA: conceptualization, resources, supervision, and writing—review and editing. KO: conceptualization, resources, supervision, funding acquisition, and writing—review and editing. All authors contributed to the article and approved the submitted version. The author contributions were described according to CRediT (Contributor Roles Taxonomy) terms (94).
