# Supplementary material for: Integration of proteomic and metabolomic analyses: New insights for mapping informal workers exposed to potentially toxic elements
Source: Front Public Health. 2023 Jan 25;10:899638. doi: 10.3389/fpubh.2022.899638 (PMC9905639; doi:10.3389/fpubh.2022.899638)
Supplement: Supplementary file 1 [file Data_Sheet_1.docx]

Supplementary Material 1

# Supplementary data on Materials and Methods

## Proteomic methods

### Protein enzymatic digestion

The enzymatic digestion was performed by adding 50 µL of RapiGest surfactant 0.2% in 100 μg of extracted proteins and incubating on a heating block at 80ºC for 15 minutes. The alkylation step was performed next by adding 5 μL L^-1^ of dithiothreitol 100mmol and incubating at 60°C for 30 minutes. A total of 5μL of iodoacetamide 300mmol L^-1^ were then added and incubated at room temperature in the dark for 30 minutes. The desaturated proteins were submitted to enzymatic digestion by adding trypsin at 1:100 proportion (Promega, USA). In this step, the samples were kept overnight at 37ºC. After 18 hours, the reaction was interrupted by adding 50μL trifluoroacetic (TFA) acid 0.1% (Sigma-Aldrich, USA). The final pH of all samples was measured and adjusted to a final value < 2. Peptides were desalinated and purified on a specific column (Pierce, #87776), before LC-MS/MS analysis.

### LC/MSMS analysis

Analysis was performed on a nanoACQUITY UPLC® System coupled with a mass spectrometry system (maXis ™ 3G Q-TOF). First, samples were loaded in a trap column (nanoACQUITY UPLC® 2G-V/MTrap 5 µm Symmetry® C18 180 µm x 20 mm, Waters, EUA). The column temperature was maintained at 40 ºC and samples were separated using a shifting gradient phase consisting of (A) 0.1% formic acid in water and (B) 0.1% of formic acid in acetonitrile, at a flow rate of 0.3 µL/min and injection volume of 2 µL. Peptides were separated using a linear gradient of 0–2% mobile phase B (1 min), 2–30% B (239 min), 30–35% B (5 min), 35-85% B (5 min), 85% B (5 min), 85–2% B (5 min), and 2% B (5 min). Eluted peptides were then ionized through a Captive Spray (Bruker Daltonics, Germany) in a maXis™ 3G Q-TOF mass spectrometer (Bruker Daltonics, Germany). The run was performed in positive mode at a desolvation temperature of 150 ºC and drying gas (N_2_) flow rate of 3 L/min. Electrospray nebulization took place at 2.0 KV and 0.4 Bar.

### Details on protein identification

Number of cleavages lost: 3; Fixed changes: Carbamidomethyl (C); Variable modifications: Oxidation (M); Peptide tolerance: 0.1 Da; Fragment tolerance: 0.1 Da; Ion charge: (1 +, 2 +, 3 +); Mass values: Monoisotopic; Instrument: ESI-QUAD-TOF (1,2).

## Metabolomics

Plasma samples were prepared by precipitating the proteins with MetOH/EtOH (1:1) (3,4). Analysis was performed on a liquid chromatography system (Agilent 1290 Infinity II UHPLC, Agilent Technologies) coupled with a QqTOF high resolution mass spectrometer (Impact HD, Bruker Daltonics GmbH, Germany) equipped with an ESI ionization source. The samples were analyzed in positive and negative ionization modes for an m/z range of 50-1300. The ionization conditions were applied as follows: capillary voltage of 500-4500V, nebulizer gas pressure of 4.0 bar, drying gas flow of 8 L/min and solvation temperature of 180 °C. In the present study, only reversed-phase liquid chromatography (RPLC) was conducted, limiting the number of polar metabolites identified. Nevertheless, the metabolites found vary for many different reasons, such as the extraction protocol, column and technique used (GC-MS, CE-MS). All of these factors can influence the metabolomic non-target assessment but, in our experience (5,6), the metabolic pathways affected tend to be the same. Considering we conducted an exploratory study and the primary goal was to assess the modified pathways, the RPLC technique was adopted in line with other studies (7). Our primary goal was mapping the metabolic disruptions in response to the chemical exposures. Therefore, the believe the use of a non-targeted approach to obtain a non-guided mapping (8), namely, RPLC with C18 column and two ionization modes (positive and negative), together with chemometric tools, disclosed sufficient metabolic pathways to biochemically explain the research question. Finally, Data were converted to mxML format using MSConvert and processed with XCMS 3.12.0 software (9–11) using R 4.0.5 and RStudio interfaces.

## Statistical analysis and bioinformatics

Pathway enrichment analysis was performed using Mummichog (version: 2.1.1-beta-20180623, using metabolic model MFN_1.10.4). Mummichog parameters were set to match against ions included in the ‘positive mode’ setting at ± 8 ppm mass tolerance ("M+H[1+]" and "M+Na[1+]").

Mummichog assigned tentative annotations to 196 of the 1055 features as significant (P<0.05) for the exposure and control groups of the total population. According to Mummichog, Empirical Compounds are putative metabolites as measured by Liquid chromatography coupled with high-resolution mass spectrometry (LC-HRMS). These putative metabolites can contain a mixture of enantiomers, stereoisomers and positional isomers that are not resolved by the instruments (12).

Additionally, to further validate the pathways proposed by Mummichog, manual curation of the metabolite identities assigned by Mummichog was carried out. For the compounds previously identified by the laboratory and for which pure chemical standards were available, retention times were compared to exclude false Mummichog annotations. The results showed that the three enriched pathways with overlap size ≥4 for the overall population “Purine metabolism”, “Valine, leucine and isoleucine degradation” and “Aspartate and asparagine metabolism” (Supplementary Table 1).

For the subsample population of the 26 participants (13 welders and 13 control group), the results showed that the three enriched pathways with overlap size ≥4 were Arginine and Proline Metabolism, Carnitine shuttle, Purine metabolism, Urea cycle/amino group metabolism, Glycerophospholipid metabolism, Valine, leucine and isoleucine degradation, Aspartate and asparagine metabolism, Vitamin E metabolism, Prostaglandin formation from arachidonate, Pyrimidine metabolism, Glycine serine alanine and threonine metabolism (Supplementary Table 2).

**Supplementary Table 1.** Mummichog analysis of statistically significant pathways for exposure and control group of the total population

**Supplementary Table 2.** Mummichog analysis of statistically significant pathways for 13 welders and 13 control participants.

# References

1. Benjamini Y, Hochberg Y. Controlling the False Discovery Rate: A Practical and Powerful Approach to Multiple Testing. *Journal of the Royal Statistical Society: Series B (Methodological)* (1995) 57:289–300. doi: 10.1111/j.2517-6161.1995.tb02031.x

2. Wenger CD, Coon JJ. A Proteomics Search Algorithm Specifically Designed for High-Resolution Tandem Mass Spectra. *J Proteome Res* (2013) 12:1377–1386. doi: 10.1021/pr301024c

3. Dudzik D, Zorawski M, Skotnicki M, Zarzycki W, Kozlowska G, Bibik-Malinowska K, Vallejo M, García A, Barbas C, Ramos MP. Metabolic fingerprint of Gestational Diabetes Mellitus. *J Proteomics* (2014) 103:57–71. doi: 10.1016/j.jprot.2014.03.025

4. Ciborowski M, Javier Rupérez F, Martínez-Alcázar MP, Angulo S, Radziwon P, Olszanski R, Kloczko J, Barbas C. Metabolomic Approach with LC−MS Reveals Significant Effect of Pressure on Diver’s Plasma. *J Proteome Res* (2010) 9:4131–4137. doi: 10.1021/pr100331j

5. Furtado DZS, de Moura Leite FBV, Barreto CN, Faria B, Jedlicka LDL, de Jesus Silva E, da Silva HDT, Bechara EJH, Assunção NA. Profiles of amino acids and biogenic amines in the plasma of Cri-du-Chat patients. *J Pharm Biomed Anal* (2017) 140:137–145. doi: 10.1016/j.jpba.2017.03.034

6. Araújo BR, Furtado DZS, de Moura Leite FBV, de Assunção NA, Carrilho E. Metabolic profiling of organic acids in urine samples of Cri Du Chat syndrome individuals by gas chromatography-mass spectrometry. *Journal of Chromatography B* (2020) 1153:122267. doi: 10.1016/j.jchromb.2020.122267

7. Gika HG, Theodoridis GA, Plumb RS, Wilson ID. Current practice of liquid chromatography–mass spectrometry in metabolomics and metabonomics. *J Pharm Biomed Anal* (2014) 87:12–25. doi: 10.1016/j.jpba.2013.06.032

8. Vinayavekhin N, Saghatelian A. “Untargeted Metabolomics.,” *Current Protocols in Molecular Biology*. Hoboken, NJ, USA: John Wiley & Sons, Inc. (2010) doi: 10.1002/0471142727.mb3001s90

9. Smith CA, Want EJ, O’Maille G, Abagyan R, Siuzdak G. XCMS:  Processing Mass Spectrometry Data for Metabolite Profiling Using Nonlinear Peak Alignment, Matching, and Identification. *Anal Chem* (2006) 78:779–787. doi: 10.1021/ac051437y

10. Tautenhahn R, Böttcher C, Neumann S. Highly sensitive feature detection for high resolution LC/MS. *BMC Bioinformatics* (2008) 9:504. doi: 10.1186/1471-2105-9-504

11. Benton HP, Want EJ, Ebbels TMD. Correction of mass calibration gaps in liquid chromatography–mass spectrometry metabolomics data. *Bioinformatics* (2010) 26:2488–2489. doi: 10.1093/bioinformatics/btq441

12. Pang Z, Chong J, Li S, Xia J. MetaboAnalystR 3.0: Toward an Optimized Workflow for Global Metabolomics. *Metabolites* (2020) 10:186. doi: 10.3390/metabo10050186

13. Jacob Cohen. *Statistical Power Analysis for the Behavioral Sciences*. Routledge (2013).

14. Stephane Champely, Claus Ekstrom, Peter Dalgaard, Jeffrey Gill, Stephan Weibelzahl, Aditya Anandkumar, Clay Ford, Robert Volcic, Helios De Rosario. Package ‘pwr’. R package version 1(2). (2018)

# Tables

**Supplementary Table 1.** Mummichog analysis of statistically significant pathways for exposure and control group of the total population

**Supplementary Table 2.** Mummichog analysis of statistically significant pathways for 13 welders and 13 control participants.

**Supplementary Table 3**. Descriptive Statistics

**Supplementary Table 4**. Gene ontology of significant proteins upregulated in welder group. Limeira, São Paulo, 2017

**Supplementary Table 5**. Gene ontology of significant proteins downregulated in welder group. Limeira, São Paulo, 2017

**Supplementary Table 6**. Blood PTE levels (mean and standard deviation, µgL^-1^) by exposure group for proteomic analysis. Limeira, São Paulo, Brazil, 2017

**Supplementary Table 7**. Blood PTE levels (mean and standard deviation, µgL^-1^) by exposure group for metabolomic analysis. Limeira, São Paulo, Brazil, 2017

**Supplementary Table 8** - Correlation of gene saliva expression (Feature1) associated with blood Ni, Cu, Zn, Sn, Sb and Pb metal concentrations, (Feature2, mean)

# Figures

*
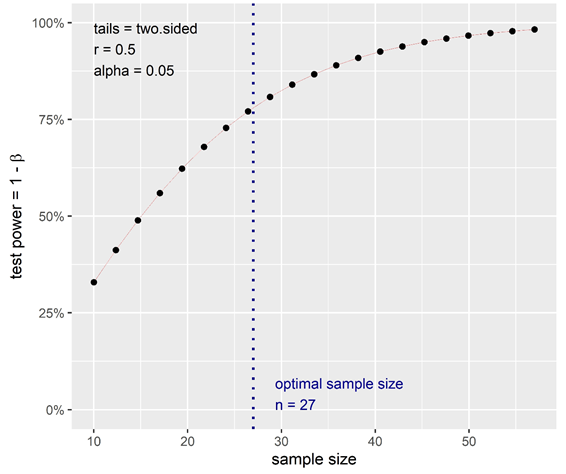
*

**Supplementary Figure 1.** Calculated statistical power (13) (Power; solid red line) for significance level 0.05 and effect size of 0.5. The blue dot line indicates a sample size of 27 participants whose statistical power is 0.8. The analysis was carried out with the R pwr package (14).


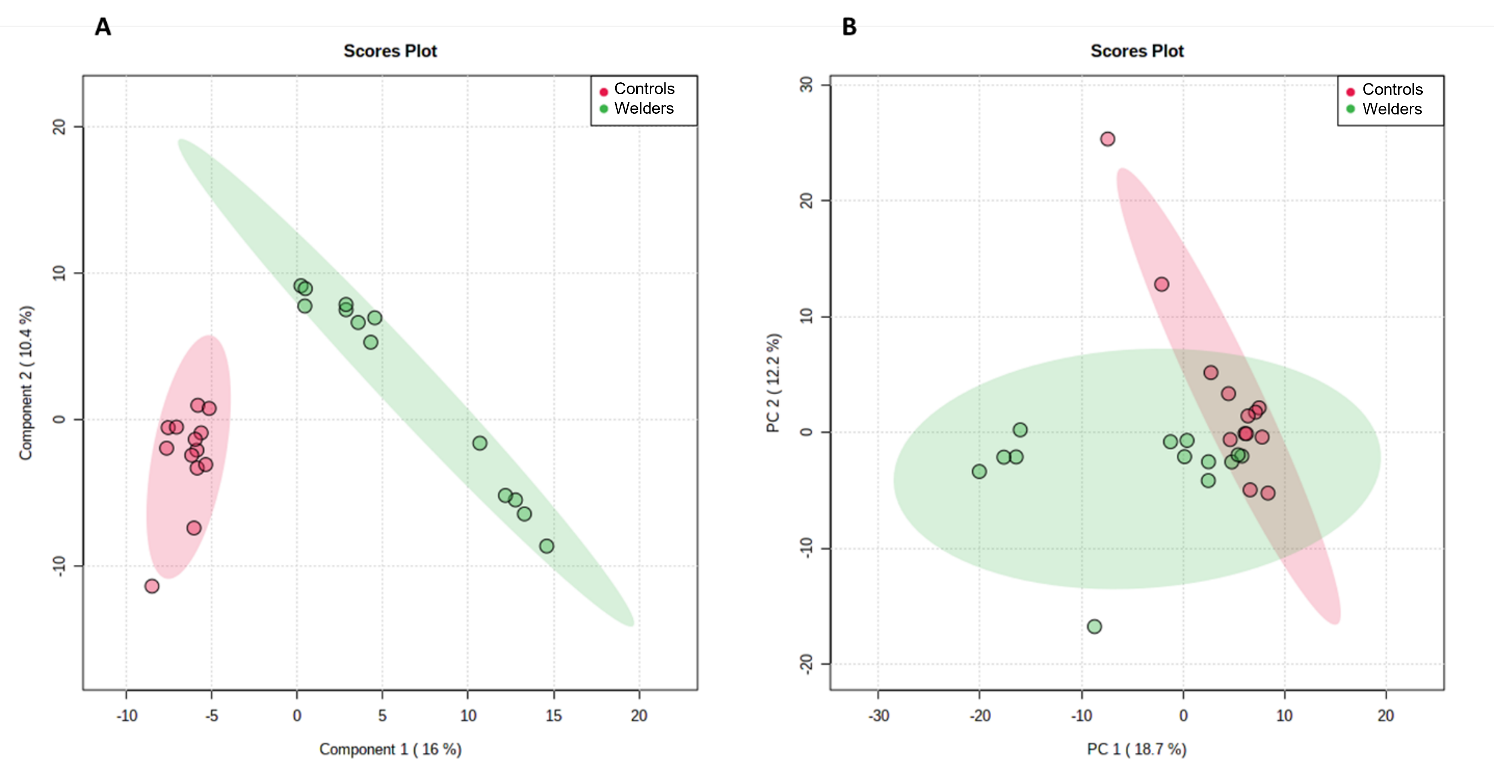


**Supplementary Figure 2.** PCA and PLS-DA score plots.
