# Supplementary material for: Integration of proteomic and metabolomic analyses: New insights for mapping informal workers exposed to potentially toxic elements
Source: Front Public Health. 2023 Jan 25;10:899638. doi: 10.3389/fpubh.2022.899638 (PMC9905639; doi:10.3389/fpubh.2022.899638)
Supplement: Supplementary file 2 [file Table_1.docx]

**Supplementary Table 1.** Mummichog analysis of statistically significant pathways for exposure and control group of the total population

| **Pathways** | Overlap size^A^ | Pathway size^B^ | p-value^C^ | KEGG compounds^D^ |
| --- | --- | --- | --- | --- |
| Purine metabolism | 5 | 10 | 0.00042 | C00294, C01620/C00262, C00402/C00049, C01620/C00262, C00294 |
| Valine, leucine, and isoleucine degradation | 4 | 7 | 0.00084 | C00719/C00183/C00431, C06102/C03465/C00671/C00233, CE5068, C00407/C00123 |
| Aspartate and asparagine metabolism | 4 | 16 | 0.04176 | C05936/C00408, C00402/C00049, C03078, C02238/C01879/C04281/C04282 |

^A^ Pathway size is number of detected Empirical Compounds for each pathway.

^B^ Overlap size is number of significant Empirical Compounds.

^C^ Empirical p-values are estimated by permutation test.

^D^ Details on empirical compounds are available in Supplementary Material (1.3).
