# Supplementary material for: Integration of proteomic and metabolomic analyses: New insights for mapping informal workers exposed to potentially toxic elements
Source: Front Public Health. 2023 Jan 25;10:899638. doi: 10.3389/fpubh.2022.899638 (PMC9905639; doi:10.3389/fpubh.2022.899638)
Supplement: Supplementary file 3 [file Table_2.docx]

**Supplementary Table 2.** Mummichog analysis of statistically significant pathways for 13 welders and 13 control participants

| **Pathways** | Overlap size^A^ | Pathway size^B^ | p-value^C^ | KEGG compounds^D^ |
| --- | --- | --- | --- | --- |
| Arginine and Proline Metabolism | 8 | 19 | 0.00156 | C00148, C05947, C00022, C04188, C03564, C03912, C04582, C00763, C00049, C05936 |
| Carnitine shuttle | 5 | 11 | 0.00299 | hdcecrn, c226crn, pcrn, strdnccrn, hdd2crn |
| Purine metabolism | 6 | 15 | 0.00351 | C05512, C00294, C00022, C00049, C01551, C00499 |
| Urea cycle/amino group metabolism | 6 | 15 | 0.00351 | C00148, C00624, C01586, C03912, C00049, C05936 |
| Glycerophospholipid metabolism | 5 | 13 | 0.00644 | C00836, C06893, C00588, C05650, C00222 |
| Valine, leucine and isoleucine degradation | 6 | 17 | 0.00688 | CE5068, C00407, C00671, C00123, C03465, C00233, C00183 |
| Aspartate and asparagine metabolism | 8 | 16 | 0.01310 | C00148, C03078, C05947, C00402, C03413, CE4788, C00049, C02630, C05936 |
| Vitamin E metabolism | 6 | 20 | 0.01848 | CE5856, CE5847, CE5723, CE7144, CE5855, CE4898 |
| Prostaglandin formation from arachidonate | 7 | 26 | 0.03087 | C00425, C01312, C00427, C05962, CE5708, C00584, C00696, C05956, C11695, C02198, CE0955 |
| Pyrimidine metabolism | 4 | 13 | 0.03098 | C00906, C00049, C00178, C00022 |
| Glycine, serine, alanine and threonine metabolism | 4 | 14 | 0.04376 | C02218, C03508, C00719, C00022 |

^A^ Pathway size is number of detected Empirical Compounds for each pathway.

^B^ Overlap size is number of significant Empirical Compounds.

^C^ Empirical p-values are estimated by permutation test.

^D^ Details on KEGG compounds are available in Supplementary Material 2.
