# Supplementary material for: Integration of proteomic and metabolomic analyses: New insights for mapping informal workers exposed to potentially toxic elements
Source: Front Public Health. 2023 Jan 25;10:899638. doi: 10.3389/fpubh.2022.899638 (PMC9905639; doi:10.3389/fpubh.2022.899638)
Supplement: Supplementary file 4 [file Table_3.docx]

**Supplementary Table 3**. Descriptive Statistics

| Description | **Metabolomics** | | | **Proteomics** | | |
| --- | --- | --- | --- | --- | --- | --- |
|  | Exposure  (n=97) | Control  (n=48) | Overall (n=145) | Exposure (n=13) | Control (n=13) | Overall  (n=26) |
| **Age (years)** |  |  |  |  |  |  |
| Median (Min-Max) | 25 (2-86) | 35 (3-71) | 28 (2-86) | 38 (19-65) | 41 (21-70) | 39 (19-70) |
| **Sex (n)** |  |  |  |  |  |  |
| Male | 42 (43.3%) | 14 (29.2%) | 56 (38.6%) | 0(0%) | 0(0%) | 0(0%) |
| Female | 55 (56.7%) | 34 (70.8%) | 89 (61.4%) | 13(100%) | 13(100%) | 26(100%) |
| **Exposure occupation** (n) |  |  |  |  |  |  |
| Performs welding | 28 (28.9%) | 0(0%) | 28 (19.3%) | 13 (100%) | 0(0%) | 13(50%) |
| Performs assembly only | 25 (25.7%) | 0(0%) | 25 (17.2%) | 0(0%) | 0(0%) | 0(0%) |
| Relative of worker (not engaged in jewelry work) | 44 (45.4%) | 0(0%) | 44 (30.3%) | 0(0%) | 0(0%) | 0(0%) |
