# Supplementary material for: Integration of proteomic and metabolomic analyses: New insights for mapping informal workers exposed to potentially toxic elements
Source: Front Public Health. 2023 Jan 25;10:899638. doi: 10.3389/fpubh.2022.899638 (PMC9905639; doi:10.3389/fpubh.2022.899638)
Supplement: Supplementary file 5 [file Table_4.docx]

**Supplementary Table 4**. Gene ontology of significant proteins upregulated in welder group. Limeira, São Paulo, 2017

| Expression regulated | [Term GO](https://david.ncifcrf.gov/chartReport.jsp?d-16544-p=1&d-16544-o=2&annot=30&d-16544-s=2) | [%](https://david.ncifcrf.gov/chartReport.jsp?d-16544-p=1&d-16544-o=1&annot=30&d-16544-s=6) | [P-Value](https://david.ncifcrf.gov/chartReport.jsp?d-16544-p=1&d-16544-o=1&annot=30&d-16544-s=7) |
| --- | --- | --- | --- |
| BP | GO:0006413~translational initiation | 20.0 | 2.80E-05 |
| BP | GO:0006614~SRP-dependent co-translational protein targeting to membrane | 16.0 | 2.40E-04 |
| BP | GO:0019083~viral transcription | 16.0 | 4.10E-04 |
| BP | GO:0000184~nuclear-transcribed mRNA catabolic process, nonsense-mediated decay | 16.0 | 4.80E-04 |
| BP | GO:0033206~meiotic cytokinesis | 8.0 | 2.60E-03 |
| BP | GO:0016344~meiotic chromosome movement towards spindle pole | 8.0 | 2.60E-03 |
| BP | GO:0006364~rRNA processing | 16.0 | 2.60E-03 |
| BP | GO:0006412~translation | 16.0 | 4.20E-03 |
| BP | GO:0008356~asymmetric cell division | 8.0 | 5.20E-03 |
| BP | GO:0006928~movement of cell or subcellular component | 12.0 | 5.60E-03 |
| BP | GO:0051653~spindle localization | 8.0 | 6.50E-03 |
| BP | GO:0034314~Arp2/3 complex-mediated actin nucleation | 8.0 | 2.80E-02 |
| BP | GO:0002181~cytoplasmic translation | 8.0 | 3.20E-02 |
| BP | GO:0007163~establishment or maintenance of cell polarity | 8.0 | 3.70E-02 |
| MF | GO:0003735~structural constituent of ribosome | 16.0 | 3.30E-03 |
| MF | GO:0005515~protein binding | 76.0 | 9.00E-03 |
| MF | GO:0005200~structural constituent of cytoskeleton | 12.0 | 9.70E-03 |
| MF | GO:0044822~poly(A) RNA binding | 24.0 | 1.60E-02 |
| MF | GO:0004198~calcium-dependent cysteine-type endopeptidase activity | 8.0 | 2.80E-02 |
| CC | GO:0070062~extracellular exosome | 88.0 | 1.0E-16 |
| CC | GO:0005925~focal adhesion | 40.0 | 3.40E-10 |
| CC | GO:0016020~membrane | 60.0 | 1.70E-08 |
| CC | GO:0005829~cytosol | 60.0 | 3.10E-06 |
| CC | GO:0043209~myelin sheath | 16.0 | 7.80E-04 |
| CC | GO:0005840~ribosome | 16.0 | 1.00E-03 |
| CC | GO:0031012~extracellular matrix | 16.0 | 5.20E-03 |
| CC | GO:0005885~Arp2/3 protein complex | 8.0 | 1.40E-02 |
| CC | GO:0015629~actin cytoskeleton | 12.0 | 2.80E-02 |
| CC | GO:0005764~lysosome | 12.0 | 3.00E-02 |
| CC | GO:0005769~early endosome | 12.0 | 3.10E-02 |

BP: biological process; MF: molecular function; CC: cellular component.
