# Supplementary material for: Integration of proteomic and metabolomic analyses: New insights for mapping informal workers exposed to potentially toxic elements
Source: Front Public Health. 2023 Jan 25;10:899638. doi: 10.3389/fpubh.2022.899638 (PMC9905639; doi:10.3389/fpubh.2022.899638)
Supplement: Supplementary file 6 [file Table_5.docx]

**Supplementary Table 5**. Gene ontology of significant proteins downregulated in welder group. Limeira, São Paulo, 2017

| Expression regulated | Term GO | % | P-Value |
| --- | --- | --- | --- |
| BP | GO:0038096~Fc-gamma receptor signaling pathway involved in phagocytosis | 23.1 | 1,50E-03 |
| BP | GO:0031333~negative regulation of protein complex assembly | 15.4 | 8,10E-03 |
| BP | GO:0031424~keratinization | 15.4 | 2,30E-02 |
| BP | GO:0030307~positive regulation of cell growth | 15.4 | 3,90E-02 |
| BP | GO:0006956~complement activation | 15.4 | 4,10E-02 |
| BP | GO:0006958~complement activation, classical pathway | 15.4 | 4,60E-02 |
| MF | GO:0003823~antigen binding | 15.4 | 4,80E-02 |
| CC | GO:0070062~extracellular exosome | 53.8 | 2,80E-04 |
| CC | GO:0005737~cytoplasm | 53.8 | 8,80E-03 |

BP: biological process; MF: molecular function; CC: cellular component.
