# Supplementary material for: Integration of proteomic and metabolomic analyses: New insights for mapping informal workers exposed to potentially toxic elements
Source: Front Public Health. 2023 Jan 25;10:899638. doi: 10.3389/fpubh.2022.899638 (PMC9905639; doi:10.3389/fpubh.2022.899638)
Supplement: Supplementary file 7 [file Table_6.docx]

**Supplementary Table 6**. Blood PTE levels (mean and standard deviation, µgL^-1^) by exposure group for proteomic analysis. Limeira, São Paulo, Brazil, 2017

| PTE | Welder group (n=13) | Control group (n=13) | P-value* |
| --- | --- | --- | --- |
| Ni | 6.181 (2.374) | 6.214 (1.694) | 0.9677 |
| Cu | 1374.515 (299.738) | 1005.692 (140.460) | <0.001* |
| Zn | 3375.630 (821.795) | 3498.595 (710.284) | 0.687 |
| Sn | 1.218 (0.740) | ^†^ | - |
| Sb | 1.925 (1.089) | 1.879 (0.863) | 0.907 |
| Pb | 20.211 (17.674) | 9.949 (4.251) | 0.053 |

*Statistical significance between groups.

^†^Not calculated: all results found were below limit of quantification.
