# Supplementary material for: Integration of proteomic and metabolomic analyses: New insights for mapping informal workers exposed to potentially toxic elements
Source: Front Public Health. 2023 Jan 25;10:899638. doi: 10.3389/fpubh.2022.899638 (PMC9905639; doi:10.3389/fpubh.2022.899638)
Supplement: Supplementary file 8 [file Table_7.docx]

**Supplementary Table 7**. Blood PTE levels (mean and standard deviation, µgL^-1^) by exposure group for metabolomic analysis. Limeira, São Paulo, Brazil, 2017

| PTE | Exposure group (n=97) | Control group (n=48) | P-value* |
| --- | --- | --- | --- |
| Ni | 6.07 (2.39) | 5.89 (1.83) | 0.615 |
| Cu | 1080 (283) | 997 (193) | 0.034* |
| Zn | 3370 (847) | 3370 (573) | 0.951 |
| Sn | 1.30 (1.18) | ^†^ | - |
| Sb | 1.78 (0.95) | 1.78 (0.85) | 0.979 |
| Pb | 16.8 (11.3) | 10.3 (5.8) | <0.001* |

*Statistical significance between groups.

^†^Not calculated: all results found were below the limit of quantification.
