# Supplementary material for: Integration of proteomic and metabolomic analyses: New insights for mapping informal workers exposed to potentially toxic elements
Source: Front Public Health. 2023 Jan 25;10:899638. doi: 10.3389/fpubh.2022.899638 (PMC9905639; doi:10.3389/fpubh.2022.899638)
Supplement: Supplementary file 9 [file Table_8.docx]

**Supplementary Table 8** - Correlation of gene saliva expression (Feature1) associated with blood Ni, Cu, Zn, Sn, Sb and Pb metal concentrations, (Feature2, mean).

| **Feature1** | **Feature2** | **Correlation** | **P.value** | **Statistic** |
| --- | --- | --- | --- | --- |
| **LV39** | Cu ppb | 0.744 | <0.001 | 5.451 |
| **SPR1A** | Cu ppb | 0.688 | <0.001 | 4.649 |
| **CRBG1** | Cu ppb | -0.474 | 0.015 | -2.635 |
| **LV743** | Cu ppb | 0.463 | 0.017 | 2.558 |
| **FLNB** | Cu ppb | -0.395 | 0.046 | -2.107 |
| **RAB5B** | Cu ppb | -0.392 | 0.047 | -2.090 |
| **CAN1** | Cu ppb | -0.388 | 0.050 | -2.063 |
| **ARP2** | Pb ppb | -0.489 | 0.011 | -2.743 |
| **1433G** | Pb ppb | -0.406 | 0.040 | -2.177 |
| **IF4A1** | Sb ppb | -0.421 | 0.032 | -2.277 |
| **ARF6** | Sb ppb | -0.418 | 0.034 | -2.253 |
| **RS10** | Sb ppb | -0.414 | 0.035 | -2.231 |
| **PLBL1** | Sb ppb | -0.414 | 0.035 | -2.230 |
| **RS9** | Sb ppb | -0.409 | 0.038 | -2.193 |
| **ARP2** | Sn ppb | 0.711 | <0.001 | 4.953 |
| **MNDA** | Sn ppb | 0.640 | <0.001 | 4.077 |
| **1433G** | Sn ppb | 0.611 | 0.001 | 3.778 |
| **CRBG1** | Sn ppb | 0.585 | 0.002 | 3.530 |
| **RAB5B** | Sn ppb | 0.538 | 0.005 | 3.125 |
| **SAP** | Sn ppb | 0.514 | 0.007 | 2.936 |
| **LV39** | Sn ppb | -0.496 | 0.010 | -2.801 |
| **NUCB1** | Sn ppb | 0.478 | 0.013 | 2.667 |
| **CAPZB** | Sn ppb | -0.478 | 0.014 | -2.663 |
| **HV64D** | Sn ppb | -0.464 | 0.017 | -2.565 |
| **IMB1** | Sn ppb | -0.464 | 0.017 | -2.564 |
| **ARP3** | Sn ppb | 0.454 | 0.020 | 2.494 |
| **LV743** | Sn ppb | -0.419 | 0.033 | -2.258 |
| **SPR1A** | Sn ppb | -0.418 | 0.033 | -2.257 |
| **HV459** | Sn ppb | -0.418 | 0.034 | -2.254 |
| **HV461** | Sn ppb | -0.418 | 0.034 | -2.254 |
| **HVD82** | Sn ppb | -0.418 | 0.034 | -2.254 |
| **HVD34** | Sn ppb | -0.418 | 0.034 | -2.254 |
| **DDX3X** | Sn ppb | -0.417 | 0.034 | -2.246 |
| **CDC42** | Sn ppb | -0.416 | 0.035 | -2.238 |
| **PDIA6** | Sn ppb | 0.415 | 0.035 | 2.234 |
| **CAN1** | Sn ppb | 0.412 | 0.037 | 2.215 |
| **TM11D** | Sn ppb | 0.407 | 0.039 | 2.185 |
| **ARGI1** | Sn ppb | -0.398 | 0.044 | -2.128 |
| **FLNB** | Sn ppb | 0.393 | 0.047 | 2.091 |
| **LV39** | Cu ppb | 0.744 | 0.000 | 5.451 |
